# Hypoxia-altered signaling pathways of toll-like receptor 4 (TLR4) in human corneal epithelial cells

**Published:** 2009-12-02

**Authors:** Yuko Hara, Atsushi Shiraishi, Yuichi Ohashi

**Affiliations:** 1 Department of Ophthalmology, Ehime University Graduate School of Medicine, Shitsukawa, Toon, Ehime, Japan; 2 Department of Ophthalmology and Regenerative Medicine, Ehime University Graduate School of Medicine, Shitsukawa, Toon, Ehime, Japan; 3 Department of Cell Growth and Tumor Regulation, Ehime University Graduate School of Medicine, Shitsukawa, Toon, Ehime, Japan; 4 Department of Infectious Diseases, Ehime University Graduate School of Medicine, Shitsukawa, Toon, Ehime, Japan

## Abstract

**Purpose:**

Toll-like receptor 4 (TLR4), a member of the TLR family, is an important pattern recognition molecule that plays a role in the host’s innate immune responses to lipopolysaccharide (LPS), a component of gram-negative bacteria. Contact lens wear is one of the risk factors for bacterial keratitis. The purpose of this study was to determine whether hypoxia or contact lens wear alters the TLR4 signaling pathways in human corneal epithelial cells (HCECs).

**Method:**

A simian virus 40-immortalized human corneal epithelial cell (SV40-HCEC) line was cultured under 20% O_2_ or 2% O_2_ and exposed to LPS. The expression of *TLR4*, interleukin-6 (*IL-6*), and *IL-8* was determined using a real-time reverse transcription-polymerase chain reaction (RT-PCR), enzyme-linked immunosorbent assay (ELISA), and immunoblotting. Immunoblotting was also used to determine whether the nuclear factor kappa B (NFκB) was activated in the SV40-HCEs. HCECs were obtained from 17 healthy volunteers and 18 hydrogel soft contact lens (SCL) wearers using impression cytology (IC), and the expression of the mRNA of *TLR4* was determined using real-time RT-PCR.

**Results:**

A reduction in the expression of the mRNA and protein of TLR4 was detected in SV40-HCECs cultured under hypoxic conditions. Hypoxia also attenuated both the LPS-induced expression of *IL-6* and *IL-8*, and the activation of NFκB in SV40-HCECs. The expression of the mRNA of *TLR4* was down-regulated in the HCECs of soft contact lens wearers.

**Conclusions:**

These results indicate that hypoxia attenuates the TLR4 signaling pathway in HCECs, suggesting that the increase in the susceptibility to bacterial infections under hypoxic conditions may be related to the TLR4 signaling pathways.

## Introduction

Bacterial keratitis is a serious, vision-threatening disease. Until recently, most cases of bacterial keratitis were associated with trauma or ocular surface diseases [1,2]. However, with the increase in the population of contact lens wearers, contact lens wear has become one of the major predisposing factors for microbial keratitis [3-7]. Gram-positive bacteria are the predominant microbiological organisms associated with bacterial keratitis (83% of all positive cultures), and gram-negative bacteria account for only 17% of all bacterial keratitis. However, gram-negative bacteria, mainly *Pseudomonas aeruginosa*, account for 30% of all bacterial keratitis in contact lens wearers [8]. In addition, Bourcier reported that 80.1% of bacterial keratitis cases caused by gram-negative organisms were found in contact lens wearers [8].

It was recently reported that *Pseudomonas aeruginosa* was isolated in 71% of the culture-positive cases of contact lens-related keratitis, and it was the most common isolate in Australia (44.2%) [9].

Many pathophysiological effects of contact lens wear have been reported, such as allergic, toxic, mechanical, and osmotic effects. One of the important effects of contact lens wear was the induced hypoxia and hypercapnia of the corneas [10,11].

The corneal epithelial cells are the first line of defense against invading pathogens. One of the mechanisms for this resistance is the antimicrobial components of the tear film, e.g., lactoferrin, lysozyme, mucins, and defensins [12,13].

Recent investigations of the innate immune system have suggested that toll-like receptor systems are involved in the immune system on the ocular surface [14-19].

Toll-like receptors (TLRs) are a family of innate immune-recognizing receptors that recognize the conserved structure of microbes, termed pathogen-associated molecular patterns (PAMPs). TLR4, a member of the TLR family, has been studied extensively in pathogen-mediated host responses, and it functions as a primary detector of lipopolysaccharide (LPS), a component of gram-negative bacteria. Activation of TLR4 induces inflammatory responses by initiating multiple intracellular signaling events, including the activation of nuclear factor kappa B (NF-κB), which ultimately leads to the synthesis and release of many proinflammatory mediators and adhesion molecules, such as interleukin-1 (IL-1), IL-6, IL-8, tumor necrosis factor-α (TNF-), and intercellular adhesion molecule 1 (ICAM-1). On the ocular surface, TLR4 with a cluster of differentiation 14 (CD14) and LPS-binding protein (LBP) was reported to induce immune responses against infiltrating gram-negative bacteria [17,20].

The purpose of this study was to determine whether hypoxia is involved in the activation of the TLR 4 signaling systems in human corneal epithelial cells (HCECs), in the LPS-induced expressions of TLR 4, and in the release of inflammatory cytokines as well as activation of NF-κB. To accomplish this, experiments were conducted on a simian virus 40-immortalized human corneal epithelial cell (SV40-HCEC) line under normoxic and hypoxic conditions. We also examined the expression of the mRNA of TLR4 in the HCECs of hydrogel soft contact lens (SCL) wearers.

## Methods

### Human subjects

All procedures on human subjects were performed in accordance with the tenets of the principles of the Declaration of Helsinki [21]. The experimental protocol for these experiments was approved by the Institutional Review Board of Ehime University. Informed consent was obtained from all subjects after an explanation of the purpose of the study and the procedures to be used.

### Impression cytology (IC)

HCECs were collected from 17 healthy volunteers (average age, 34.4±6.4 years) and 18 SCL wearers (average age, 29.9±9.4 years) using impression cytology (IC) with informed consent. Briefly, a drop of 1% oxybuprocaine hydrochloride (Santen, Osaka, Japan) was dropped on the eye, and a 3×5 mm pre-autoclaved nitrocellulose membrane (Millipore, Bedford, MA) was placed on the cornea for 10 s. The membrane was gently removed and placed directly into 350 μl of RLT Buffer (Qiagen, Valencia, CA) for RNA extraction.

### Cell cultures

SV40-HCECs were grown to 100% confluence in a supplemented hormonal epithelial medium consisting of Dulbecco's modified eagle medium (DMEM; low glucose)/F-12 (Invitrogen, Carlsbad, CA), 15% fetal bovine serum (FBS), 10 ng/ml epidermal growth factor, 5 µg/ml insulin, 5 mM L-glutamine, 0.5% dimethyl sulfoxide, and gentamicin [22]. All cells were grown at 37 °C in a humid environment containing 5% CO_2_. The cell culture medium was changed every 2 to 3 days.

After the cells reached confluence, the SV40-HCECs were maintained in a keratinocyte serum-free medium (KSFM; Invitrogen) supplemented with 5 ng/ml of human recombinant epidermal growth factor (Invitrogen).

To analyze the effect of oxygen on cell behavior, one group of cells was maintained at 37 °C and 5% CO_2_ in a conventional humid tissue culture incubator (20% O_2_). A second group of SV40-HCECs was cultured at 37 °C in 5% CO_2_ and 2% O_2_ using an oxygen monitor to regulate the flow of a calibrated mixture of 95% N_2_ and 5% CO_2_. After 48 hours, the SV40-HCECs were exposed to 500 ng/ml of recombinant human sCD14 (R&D Systems), 150 ng/ml of recombinant human LBP (R&D Systems), and 100 ng/ml of LPS derived from *Pseudomonas aeruginosa* (Sigma, St. Louise, MO) for 24 h. The SV40-HCECs and the culture supernatant were then collected for further examination.

### Real-time reverse transcription-polymerase chain reaction (real-time PCR) analysis

Total RNA was extracted using an RNeasy kit (Qiagen, Valencia, CA) and then reverse-transcribed using Omniscript Reverse Transcriptase (Qiagen) according to the manufacturer’s protocols. Real-time PCR was performed with a DyNAmo STBR Green qPCR kit (FINNZYMES, Espoo, Finland) as follows: preheat at 95 °C for 15 min, 40 cycles of denaturation at 95 °C for 10 s, annealing at 60 °C for 20 s, and extension at 72 °C for 30 s using an OPticon2 DNA Engine (BIO RAD, Hercules, CA). The primer pairs used for real-time PCR are listed in Table 1. The C_t_ values were determined using the Opticon2 software, and the amount of each mRNA was calculated relative to the amount of *β-actin* mRNA in the same samples [23]. Each run was completed with a melting curve analysis in order to confirm the specificity of amplification and lack of primer dimmers.

**Table 1:** Primer pairs for real time PCR.

**Gene**	**Forward primer**	**Reverse primer**	**Product size (bp): Accession number**
*IL-6*	TACCCCCAGGAGAAGATTCC	TTTTCTGCCAGTGCCTCTTT	175 : M29150
*IL-8*	GTGCAGTTTTGCCAAGGAGT	CTCTGCACCCAGTTTTCCTT	196 : BC013615
*TLR4*	TGAGCAGTCGTGCTGGTATC	CAGGGCTTTTCTGAGTCGTC	167 : NM_138554
*β-actin*	GCACCACACCTTCTACAATGAG	ATAGCACAGCCTGGATAGCAAC	164 : NM_001101

### Enzyme-linked immunosorbent assay (ELISA)

The concentrations of IL-6 and IL-8 in the supernatant of the cultured SV40-HCEs were determined with an ELISA test kit (R&D Systems, Minneapolis, MN) following the manufacturer’s protocols.

### Immunoblotting

Proteins were extracted from the SV40-HCEs using an M-PER mammalian protein extraction reagent (Pierce, Rockford, IL). Each sample (10 μg) was then separated using sodium dodecyl sulfate polyacrylamide gel electrophoresis (SDS-PAGE) under reducing conditions (12% resolving gel) and transferred onto a nitrocellulose membrane (Amersham Biosciences, Piscataway, NJ). The membrane was then blocked for 1 h in 5% dried skim milk in Tris-buffered saline with 0.1% Tween-20 (T-TBS). TLR4 and NFκB were probed with primary antibodies to NFκB p65 (0.2 μg/ml in 3% bovine serum albumin (BSA) in T-TBS; Santa Cruz Biotechnology, Santa Cruz, CA) or TLR4 (0.2 μg/ml in 3% BSA in T-TBS, BioLegend, San Diego, CA) overnight at 4 °C. The positive immunoreactions were made visible by an enhanced chemiluminescence (ECL plus) detection system (Amersham Pharmacia Biotech). The expression levels of NFκB p65 and TLR4 were determined relative to that of β-actin in the same sample using Quantity one volume analysis (Bio Rad).

### Statistical analyses

Each experiment was repeated three times, and representative results are shown in the figures. The values are the means ±standard deviations (SDs). Differences between the groups were tested using a two-tailed paired *t* test. A p-value of <0.05 was considered to be statistically significant.

## Results

### Hypoxia down-regulated TLR4 expression

**Figure 1:**
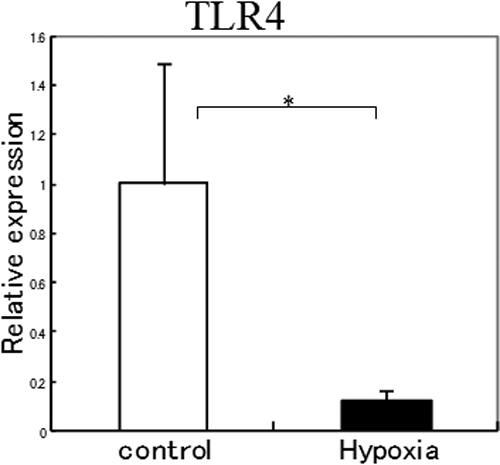
Expression of *TLR4* mRNA in SV40-HCEs cultured under 20% O_2_ (control group) or 2% O_2_ (hypoxia group). The expression of the mRNA of *TLR4* decreased to about 10% of the control group under hypoxic conditions. The p-values were calculated using two-tailed paired *t* tests. The asterisk indicates a p<0.05.

**Figure 2:**
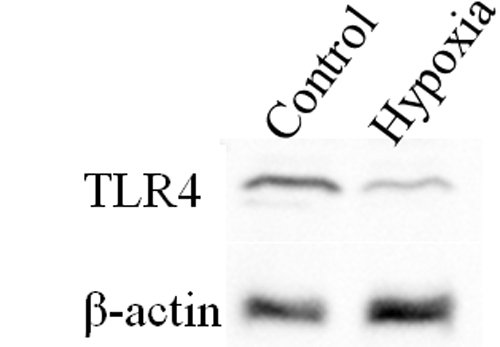
Immunoblotting for TLR4 protein expression in SV40-HCECs cultured under 20% O_2_ (control group) or 2% O_2_ (hypoxia group). TLR4 protein expression of the hypoxia group decreased to about 30% of the control group.

To determine whether the expression of TLR4 in SV40-HCECs was altered under hypoxic conditions, SV40-HCEs were cultured under 20% O_2_ (control group) or 2% O_2_ (hypoxia group) for 48 h. The expression of TLR4 was measured using real-time PCR and immunoblotting. Our results showed that the expression of the mRNA of *TLR4* in the hypoxia group had decreased to about 10% of the control group (Figure 1). Immunoblotting for the expression of the TLR4 protein in the hypoxia group was about 28% of the control group (Figure 2).

### Effect of hypoxia on the TLR 4 signaling pathway

To determine whether hypoxic conditions alter the LPS-induced cytokine/chemokines expression in SV40-HCECs, SV40-HCECs were exposed to100 ng/ml of LPS derived from *Pseudomonas aeruginosa* and co-incubated with 500 ng/ml of recombinant human sCD14 and150 ng/ml of recombinant human LBP under 20% O_2_ or 2% O_2_ for 24 h. The cells and supernatants were collected, and the expressions of the mRNAs of *IL-6* and *IL-8* were evaluated using real-time PCR. The protein levels of IL-6 and IL-8 were determined using ELISA.

**Figure 3:**
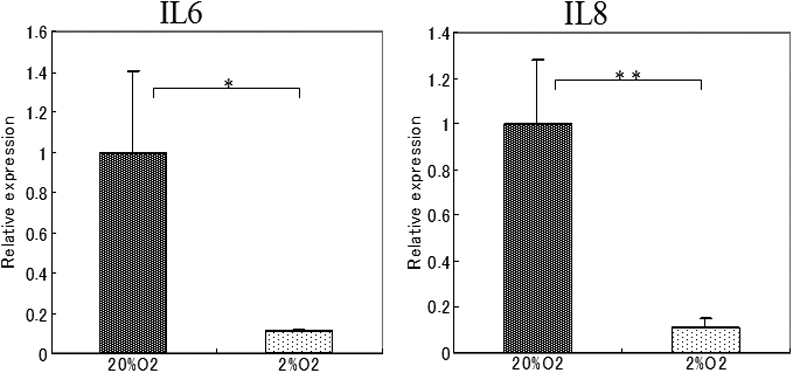
Effects of hypoxia on the expression of cytokines in SV40-HCECs exposed to LPS. Total RNA was isolated from SV40-HCECs cultured under 20% O_2_ (control group) or 2% O_2_ (Hypoxia group) for 72 h and stimulated with LPS for 24 h. The expression of the mRNA of *IL-6* and *IL-8* was determined using real-time PCR. The relative level of mRNA expression for each cytokine is normalized to *G3PDH* mRNA expression. The p-values were calculated using a two-tailed paired *t* test. The asterisk indicates a p<0.05 and the double asterisk indicates a p<0.01).

**Figure 4:**
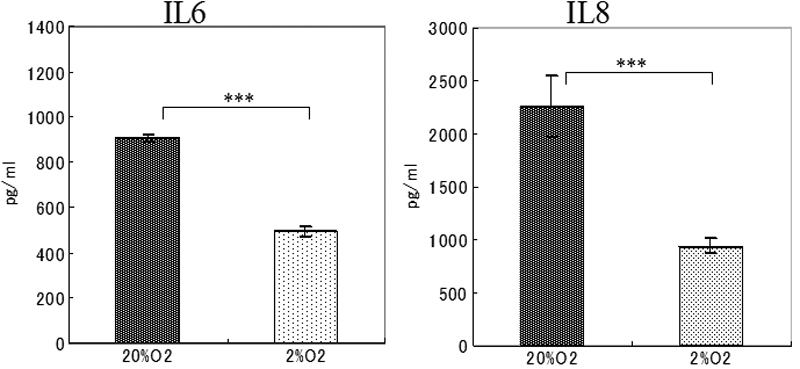
Cytokine secretion by SV40-HCEs cultured under 20% O_2_ (control group) or 2% O_2_ (hypoxia group) stimulated with LPS. The culture medium was collected 24 h after LPS exposure and analyzed for the presence of IL-6 and IL-8 proteins using ELISA. The p-values were calculated using a two-tailed paired *t* tests. The triple asterisk indicates a p<0.001).

Our results showed that the expression of the mRNA of *IL-6* in the hypoxia group had decreased to about 11% of the control group, and the *IL-8* mRNA had also decreased to about 10% of the control group (Figure 3). ELISA showed that the LPS-induced IL-6 production had decreased by about 54% under 20% O_2_, and IL-8 had decreased by about 41% under 2% O_2_ (Figure 4).

### Immunoblotting for NFκB

**Figure 5:**
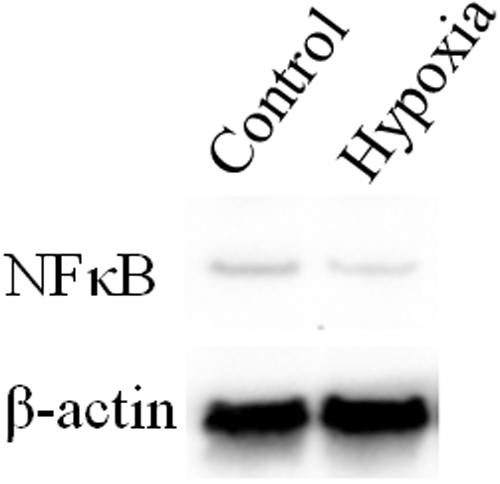
Immunoblotting for NFκB 24 h after exposure to LPS. Immunoblotting showed that NFκB protein expression in the hypoxia group was reduced compared to that in the control group.

The effect of hypoxia on the activation of NFκB was determined by immunoblotting 24 h after stimulation by LPS. Immunoblotting showed that the expression of the NFκB protein in the hypoxia group was about 48% of that in the control group (Figure 5).

### Expression of TLR4-specific mRNA in human corneal epithelial cells from normal volunteers and SCL wearers

**Figure 6:**
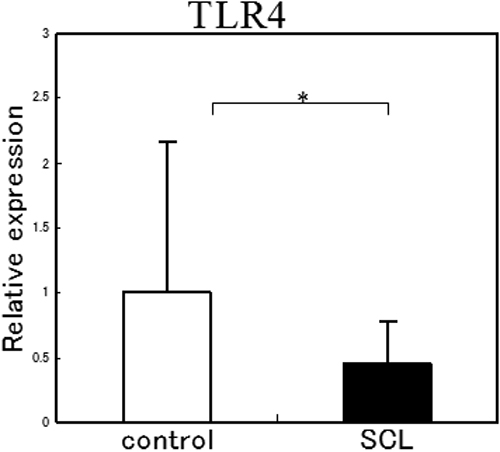
Expression of *TLR4* mRNA in the CECs of normal volunteers and SCL wearers. *TLR4* mRNA was detected from all samples, but the expression of *TLR4* from SCL wearers was one-half the amount from normal volunteers. The p-values were calculated using a two-tailed paired *t* test. The asterisk indicates a p<0.05.

We next examined whether the expression of the mRNA of *TLR4* was altered in the HCECs of SCL wearers. HCECs were collected using IC from SCL wearers and normal volunteers, and then subjected to real-time PCR. The mRNA of *TLR4* was detected in all samples, but the expression level of *TLR4* from the SCL wearers was about one-half of that in normal volunteers (Figure 6).

## Discussion

Hypoxia is considered to be one of the main risk factors for bacterial keratitis due to contact lens wear [10,11]. We hypothesized that the altered expression of the mRNA of *TLR4* in HCECs was most likely due to the hypoxia induced by the contact lens wear. Our findings demonstrated that HCECs and SV40-HCECs express the mRNA of *TLR4*, and the level of expression was decreased in SV40-HCECs cultured under hypoxic conditions. We also demonstrated that the expression of *TLR4* mRNA was decreased in the HCECs of SCL wearers. These findings are in agreement with studies reporting that *TLR4* expression was reduced by hypoxia in other type of cells and organs, e.g., cultured pulmonary artery endothelial cells [24]. On the other hand, the mRNA and protein levels of *TLR4* were up-regulated by hypoxia in a cultured microglia cell line [25]. In murine bone marrow-derived dendritic cells, hypoxia did not change the *TLR4* mRNA expression [26]. These different responses of *TLR4* expression under hypoxic conditions may be because of the different hypoxic exposure times in the different experiments. Ock et al. [25] reported that the up-regulation of *TLR4* expression in microglia was observed after 8 h of hypoxic exposure. On the other hand, Ishida et al. [24] reported that long-term (48–72 h) hypoxic exposure caused a down-regulation in the expression of *TLR4* in cultured pulmonary artery endothelial cells. Their results were in good agreement with our results on SV40-HCEs cultured under 2% O_2_ for 48 h prior to exposure to LPS. The differences in the types of cells and organs, and in the culture conditions, may account for the different *TLR4* expression. However, the down-regulated expressions of *TLR4* in HCECs under hypoxic conditions are consistent with some of the in vivo and in vitro findings.

We found that the LPS-induced expression of *IL-6* and *IL-8* had decreased and NFκB activation reduced under hypoxia. The TLR4 signaling pathways in corneal epithelial cells have been described, although the conclusions have been controversial. In an in vivo study, the *TLR4* mRNA expression was markedly increased in the cornea of Balb/c mice after infection by *Pseudomonas aeruginosa* [17,18]. On the other hand, there have been reports that the cytokine response induced by activation of TLR4 signaling due to LPS exposure was barely detected in corneal epithelial cells in vitro [14,15]. In a more recent study, sCD14 and LPS-binding protein (LBP), which are LPS receptor proteins, were identified in human tears and found to mediate the LPS-induced innate immune response by amplifying corneal epithelial cells [20]. In this study, NFκB activation was detected, followed by increased expression of *IL-6* and *IL-8* in SV40-HCECs when stimulated by LPS derived from *Pseudomonas aeruginosa* that was co-incubated with sCD14 and LBP. Our results supported those of Blais et al. [17] and Huang et al. [20], and indicated that the TLR4 signaling systems may play a role in corneal epithelial cells. In our culture system, LPS exposure caused a significant decrease in the mRNA expression of *IL-6* and *IL-8* protein production under hypoxic conditions. In addition, immunoblotting showed that the LPS-induced NFκB activation was also decreased due to hypoxia. Thus, hypoxia attenuated not only the *TLR4* expression, but also the LPS-induced TLR 4 signaling pathways.

It has been demonstrated that hypoxia activates many transcriptional factors, including NFκB [27,28]. Our findings are in contrast to those reported earlier. However, we investigated the effects of hypoxia on the LPS-induced NFκB activation or cytokine production, whereas the earlier studies investigated the effect of hypoxia on NFκB activation only. It has been reported that hypoxia up-regulated *TLR4* mRNA expression and enhanced the IRF-3 pathway, whereas it decreased the LPS-induced NFκB pathways in microglial cells [25]. Recently, it was reported that cigarette smoke causing hypoxia reduced *TLR4* mRNA and LPS responsiveness, and severe chronic obstructive pulmonary disease (COPD) had a more significant association with reduced *TLR4* expression than less severe disease [29]. The expression of *TLR4* may have important implications for inflammation and infection in response to pathogens.

Thus, NFκB activation and cytokine production are regulated complex interactions. It may be possible that hypoxia decreased the LPS-induced NFκB activation in the TLR4 signaling pathway, although there are some differences in duration, hypoxic exposure, and the use of different types of cells.

In conclusion, the expression of *TLR4* was decreased in the HCECs of SCL wearers and SV40-HCECs under hypoxic conditions. In addition, hypoxia decreased the LPS-induced expression of *IL-6* and *IL-8* as well as the activation of NFκB in SV40-HCECs. These results indicate that the contact lens-induced hypoxia may increase the susceptibility to bacterial infections such as *Pseudomonas aeruginosa* by altering the TLR4 signaling pathways.
